# A case of retroperitoneal fibrosis responding to steroid therapy

**DOI:** 10.1590/S1677-5538.IBJU.2016.0520

**Published:** 2017

**Authors:** Ryuta Watanabe, Akira Ozawa, Tokuhiro Iseda

**Affiliations:** 1 Department of Urology, Matsuyama Shimin Hospital, Ehime, Japan

**Keywords:** Retroperitoneal Fibrosis, Therapeutics, Steroids

## Abstract

A 69-year-old man presented at the hospital with complaints of prolonged stomach pain extending from the week prior. Enhanced computed tomography (CT) revealed a low density area in the retroperitoneal space. A radiologist diagnosed the patient with retroperitoneal fibrosis. One week later, an enhanced CT revealed an exponential increase of the low density area and slight right hydronephrosis. Upon admission, prednisolone administration was initiated at a dose of 40mg/day. The size of the retroperitoneal soft tissue mass decreased gradually. Although the dose of prednisolone was tapered to 5mg, the patient is doing well without any sign of recurrence.

## INTRODUCTION

**Figure 1:**
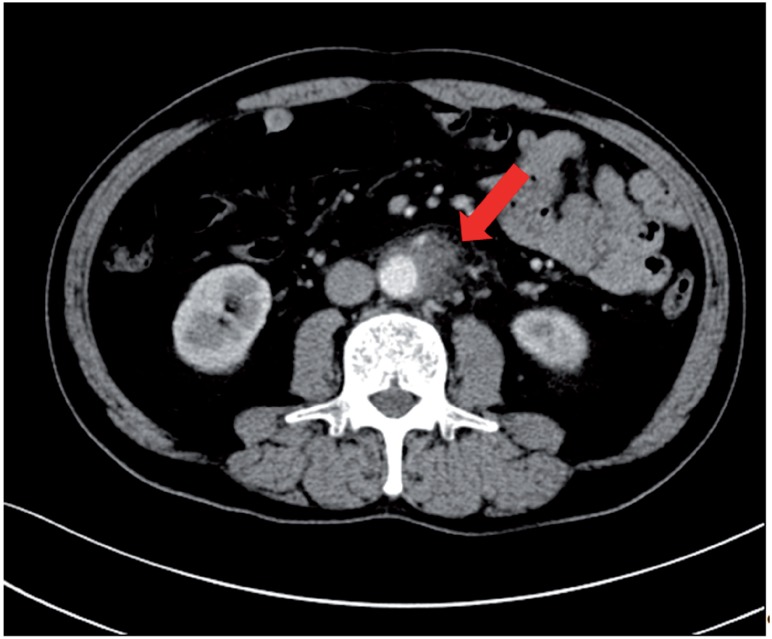
Enhanced computed tomography revealed a low density area surrounding the aorta at the level of the inferior mesenteric artery and the right total iliac artery.

A 69-year old man presented to the hospital with complaints of prolonged stomach pain, lasting from the week prior. Enhanced computed tomography revealed a low-density area surrounding the aorta at the level of the inferior mesenteric artery and the right total iliac artery (Figure-1). With a suspected dissection of the inferior mesenteric artery, the patient was taken to our hospital by ambulance. A diagnostic radiologist at our hospital re-diagnosed the case as retroperitoneal fibrosis using a previous image obtained by enhanced computed tomography (CT). The serum biochemistry exhibited elevated levels of white blood cells (WBC) and C-reactive protein (CRP) (11300/μL and 5.37mg/dL, respectively), indicating the possibility of an infection. In addition, the patient's serum creatinine level was within the normal limit (0.89mg/dL). For the purpose of observation and pain control, we administered NSAIDs and antibiotics upon admission. Since the pain was relieved by treatment with NSAIDs, the patient left the hospital momentarily the next day. However, when the patient came to the outpatient service for a follow-up study one week later, the enhanced CT revealed an exponential increase of the low-density area (Figure-2A) and slight right hydronephrosis (Figure-2B). Magnetic resonance imaging (MRI) demonstrated RPF masses to be of low to intermediate signal intensity in the T1 weighted images and low and high intensity (according to the level of inflammation) on the T2 weighted images (Figure-2C). Additionally, the patient complained once again of stomach pain and had a high fever. The patient's serum biochemistry revealed elevated levels of WBC 15000/μL, CRP 27.09mg/dL, and creatinine 1.11mg/dL; however, the serum IgG4 levels were within the normal limit (27.7mg/dL; normal range: 4.8-105). The patient was admitted once again, and prednisolone was initiated at a dose of 40mg/day under the diagnosis of retroperitoneal fibrosis with RPF aggravation. The size of the retroperitoneal soft tissue mass gradually decreased after several days (Figure-3A). Right hydronephrosis disappeared completely and the serum creatinine levels normalized. The serum CRP level also normalized on day 15 post-admission. The dose of prednisolone was tapered by 10mg every five days (Figure-4). On day 15 post-admission, the dose of prednisolone was tapered to 10mg/day. The CT revealed a remarkable reduction in the size of the retroperitoneal mass. At the time of admission, the serum levels of the soluble interleukin-2 receptor (sIL-2R) was 2990U/mL (normal range: 145-519); however, on day 19 following admission, the levels decreased to 1380U/mL. The patient was discharged on day 20. Since there was no sign of recurrence, the dose of prednisolone was tapered to 5mg/day one month after discharge. Although steroid therapy was continued for two months at a dose of 5mg/day, the CT revealed no sign of recurrence (Figure-3B). The patient is currently doing well, without any sign of recurrence.

**Figure 2:**
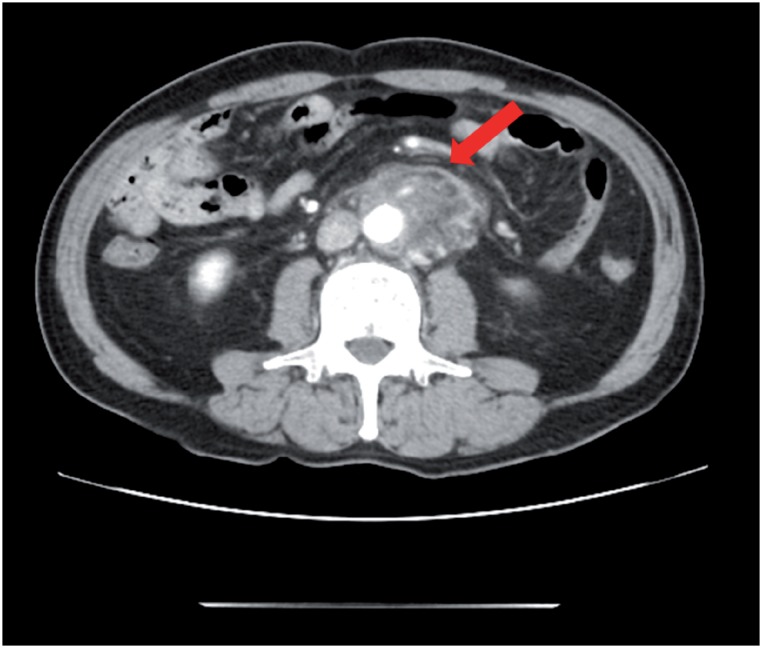
A) Enhanced computed tomography revealed an exponential increase of the low density area.

**Figure 2:**
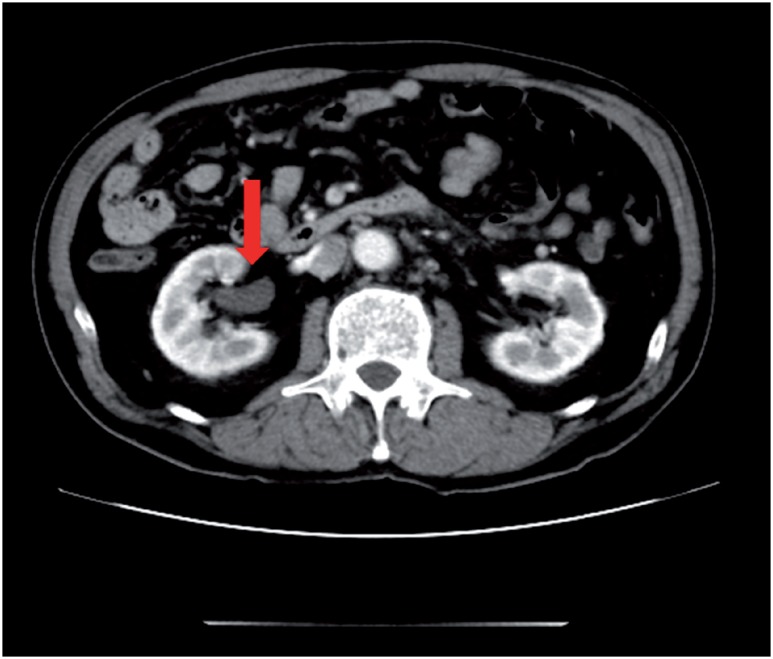
B) Enhanced computed tomography also showed a slight right hydronephrosis.

**Figure 2:**
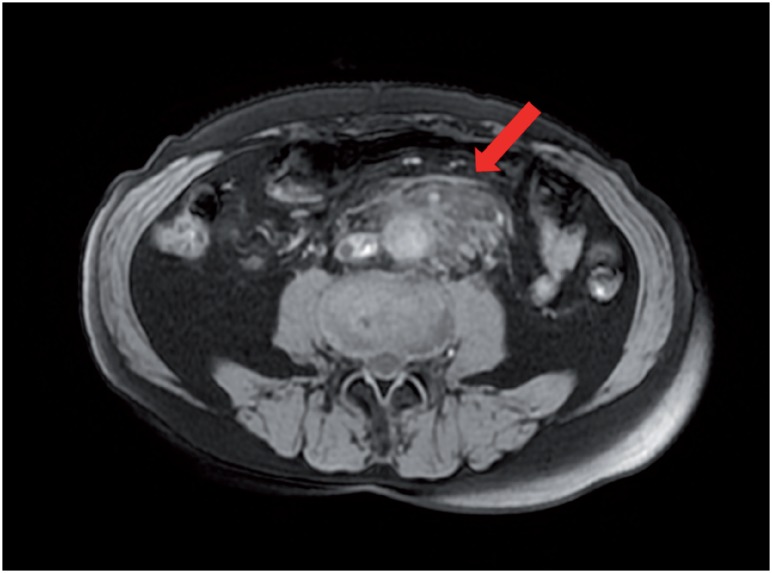
C) Magnetic resonance imaging demonstrated rpf masses as low and high intensity (according to inflammation) on the T2-weighted images.

**Figure 3:**
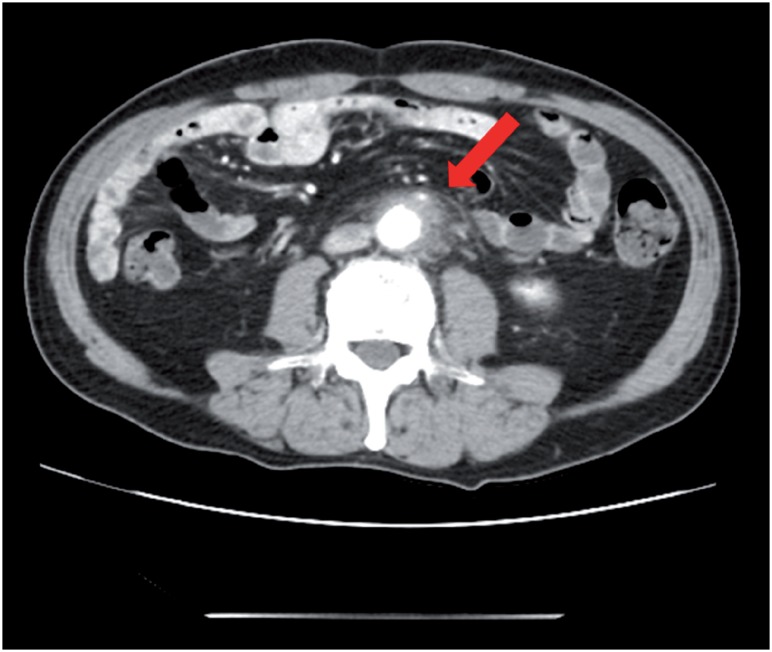
A) The size of the retroperitoneal soft tissue mass decreased on day 19 post-admission.

**Figure 3:**
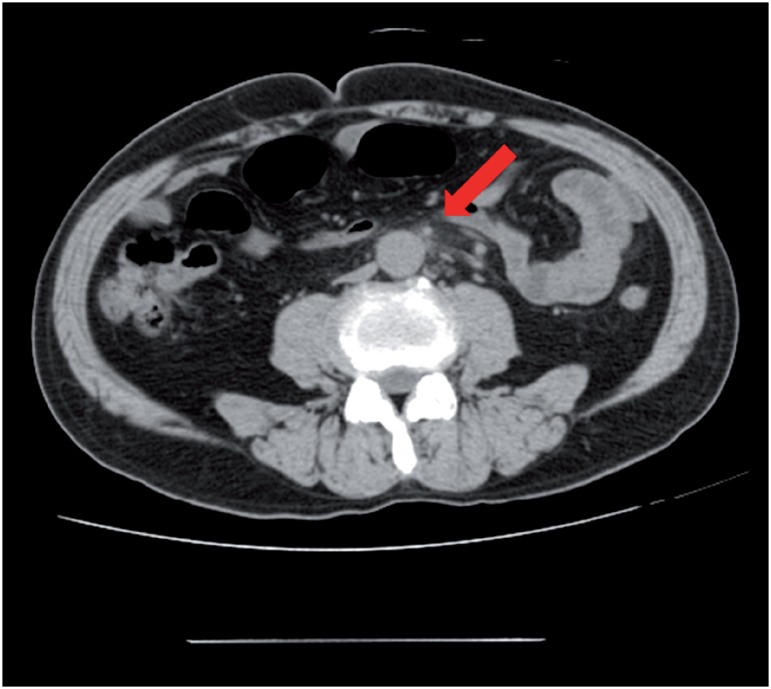
B) The size of the retroperitoneal soft tissue mass decreased two months after discharge.

**Figure 4:**
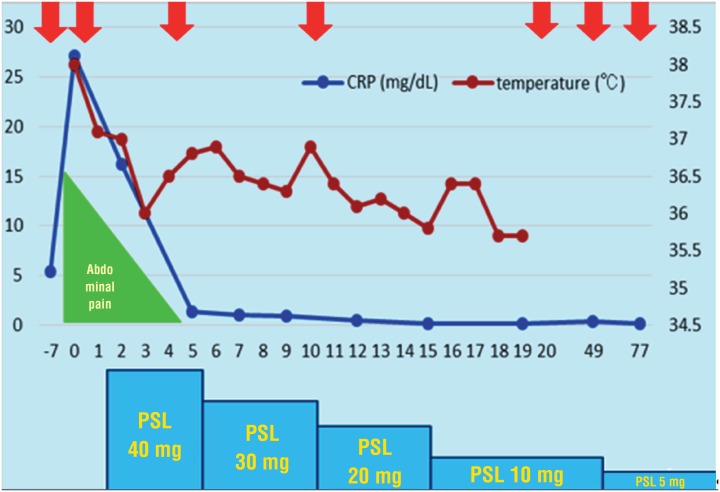
Clinical course (arrow: CT). The serum CRP level was normalized on the fifteenth admission day. The dose of prednisolone was tapered by 10 mg every five days. On day 15 after admission, the dose of prednisolone was tapered to 10 mg/day then tapered to 5 mg/day one month after discharge.

## DISCUSSION

RPF was first reported by Ormond in 1948, and has since been widely documented (3). It is a rare condition characterized by the development of peritoneal inflammation and fibrosis, which often obstructs the ureters. RPF is further categorized as either idiopathic or secondary. Idiopathic RPF encompasses IgG4-related and non-IgG-related RPF (4).

The recently recommended concept of IgG4–related disease was derived from research on autoimmune pancreatitis (AIP). AIP is characterized by the abundant infiltration of IgG4-positive plasma cells and lymphocytosis, dense fibrosis, and the presence of obliterative phlebitis in the pancreas, a pattern termed lympohplasmacytic sclerosing pancreatitis. This entity is associated with extra-pancreatic lesions exhibiting histological features similar to those of the pancreas, and is currently considered a pancreatic manifestation of IgG4-related systemic disease. RPF occasionally occurs as an extra-pancreatic lesion of AIP, and some forms of RPF can be classified as an IgG4-related disease (5).

Currently, a definitive diagnosis of IgG4-related RPF is based on the fulfillment of the following three criteria: 1) soft tissue masses surrounding the aorta and/or adjacent tissues on the CT and/or magnetic resonance imaging; 2) elevation of serum IgG4 levels (≥135mg/dL); and 3) the infiltration with IgG4-positive plasma cells (>10IgG4-positive plasma cells per high power field and a ratio of IgG4-positive to IgG-positive cells of >40:100) (6). In the present case, we could not undertake a biopsy of the retroperitoneal tissue before initiating steroid therapy due to the rapid expansion of the fibrotic area and worsening of the patient's symptoms. Moreover, while the serum IgG4 levels were normal, approximately 30% patients exhibited normal serum IgG4 levels, despite having classical histopathological and immunochemical findings (2). Our case met only one of the necessary criteria for the diagnosis of IgG4-related RPF. Thus, we could not diagnose this case as IgG4-related RPF. In addition, we diagnosed this case as non-IgG4-related or idiopathic RPF. The serum IgG4 levels have remained normal throughout the follow-up period.

Steroid therapy is recognized as the standard treatment for AIP. Therefore, steroid therapy is also strongly recommended for patients with IgG4-related RPF; however, for patients with non IgG4-related RPF, steroid therapy is also effective. Standard steroid treatment consists of an initial dose of 0.6mg/kg/day of oral prednisolone, which is reduced to a maintenance dose (5mg/day) over a period of three to six months. To prevent relapse, maintenance treatment for six months to three years is recommended (7). In the majority of cases, the pancreatic lesion or RPF improves following the initial course of treatment; however, a relapse can occur following steroid withdrawal in some cases. Our patient received standard steroid treatment resulting in almost complete remission three months after the initiation of medication. However, it is possible that we will need to increase the dose of steroids in the future due to the possibility of another recurrence. Additionally, adverse events associated with steroid therapy (e.g., gastrointestinal hemorrhage or impaired glucose tolerance) may prevent the continued use of steroid therapy. As a potential alternative to standard maintenance with steroid treatment, Fukuchi et.al. reported the efficacy and safety of Hochuekkito, a type of Kampo (i.e., traditional Japanese herbal remedy) medicine (8).

In the present case, we could not determine a definitive diagnosis prior to initiating steroid treatment. We have considered malignant lymphoma or the metastasis of malignant cancer as a differential diagnosis, but the images do not reveal any retroperitoneal nodular tumors following treatment in the area in which retroperitoneal fibrosis was present. Therefore, we therapeutically diagnosed this case as retroperitoneal fibrosis. If a case later proves to be malignant lymphoma or metastasis, we will treat for malignant lymphoma or cancer as appropriate. If we would undertake biopsy following steroid therapy, we will not be able to detect viable cells. Long term follow-up is essential for the detection of recurrence.

In the present case, the serum levels of sIL-2R were elevated, but did not provide clear evidence of malignant lymphoma because the serum levels of sIL-2R were also elevated due to increased inflammation. Moreover, the serum levels of sIL-2R decreased to 628U/mL two months after the patient was discharged.

Since we could rapidly initiate treatment with steroid therapy, we were able to avoid invasive treatment (e.g., ureter stent placement or nephrostomy) for treating hydronephrosis or renal dysfunction, although we did not clearly diagnose the situation before commencing steroid therapy. Some cases have reported that stent placement was impossible owing to strong stenosis of the ureter. Therefore, the initiation of prompt therapy is important for the treatment of retroperitoneal fibrosis (9). It is possible that the origin of the fever in this case was attributed to right hydronephrosis and pyelonephritis. However, the CT demonstrated only moderate hydronephrosis and no sign of right pyelonephritis, thus, we considered the elevated inflammatory findings and presence of fever was due to the progression of RPF. Although we could have attempted stent placement prior to the initiation of steroid therapy, it would have been difficult due to the strong stenosis of the ureter. Therefore, we decided to initiate steroid therapy immediately and carefully follow-up hydronephrosis. If the RPF progressed and hydronephrosis worsened, we would attempt a stent placement or nephrostomy. Fortunately, the right hydronephrosis disappeared rapidly by day 3 after commencing the steroid therapy.

Disease progression can be monitored by a regular evaluation of CRP and creatinine levels, as well as radiological imaging via CT, MRI, or ultrasound (to monitor hydronephrosis). The prognosis is typically good, with a relapse rate of less than 10% to 30% after discontinuing treatment (10). However, there are currently no predictors of the response to treatment or probability of relapse; thus, long-term follow-up is essential.

## CONCLUSIONS

In the present study, we report a case of retroperitoneal fibrosis responding to steroid therapy. Since we were able to rapidly begin treatment with steroid therapy, we were able to avoid invasive treatment, although we did not clearly diagnose the situation before commencing steroid therapy. Long-term follow-up by radiological imaging and blood tests are essential for detecting a recurrence.
